# A Diffusion Model Analysis of Decision Biases Affecting Delayed Recognition of Emotional Stimuli

**DOI:** 10.1371/journal.pone.0146769

**Published:** 2016-01-19

**Authors:** Holly J. Bowen, Julia Spaniol, Ronak Patel, Andreas Voss

**Affiliations:** 1 Department of Psychology, Boston College, Chestnut Hill, Massachusetts, United States of America; 2 Department of Psychology, Ryerson University, Toronto, Ontario, Canada; 3 Department of Clinical Health Psychology, College of Medicine, University of Manitoba, Winnipeg, Manitoba, Canada; 4 Psychologisches Institut, Universität Heidelberg, Heidelberg, Germany; University of Akron, UNITED STATES

## Abstract

Previous empirical work suggests that emotion can influence accuracy and cognitive biases underlying recognition memory, depending on the experimental conditions. The current study examines the effects of arousal and valence on delayed recognition memory using the diffusion model, which allows the separation of two decision biases thought to underlie memory: response bias and memory bias. Memory bias has not been given much attention in the literature but can provide insight into the retrieval dynamics of emotion modulated memory. Participants viewed emotional pictorial stimuli; half were given a recognition test 1-day later and the other half 7-days later. Analyses revealed that emotional valence generally evokes liberal responding, whereas high arousal evokes liberal responding only at a short retention interval. The memory bias analyses indicated that participants experienced greater familiarity with high-arousal compared to low-arousal items and this pattern became more pronounced as study-test lag increased; positive items evoke greater familiarity compared to negative and this pattern remained stable across retention interval. The findings provide insight into the separate contributions of valence and arousal to the cognitive mechanisms underlying delayed emotion modulated memory.

## Introduction

The effect of emotion on episodic long-term memory has been the topic of much research in psychology and neuroscience. Both the arousal and valence dimensions of emotion have been shown to affect recognition performance (for reviews, see Hamann, 2001[1]; LaBar & Cabeza, 2006[2]). However, the cognitive mechanisms underlying emotional modulation of recognition memory are still unclear. For example, some studies indicate that in addition to discriminability [3–5], decision biases are also sensitive to emotion. These include both *response bias*—the tendency to classify stimuli as “old” or “new” [6–9] and *memory bias*—the tendency to extract familiarity or novelty signals from memory [10,11]. In this context we use the term *familiarity* loosely, without specific reference to its role in dual-process models of recognition memory [12]. Furthermore, there is evidence that the emotional modulation of memory may require a period of consolidation [5,13–16], but it is not known whether emotion effects on decision biases are similarly time-dependent. The purpose of the current study was to elucidate the effects of emotional arousal and valence on response bias and memory bias at different study-test lags using diffusion modeling [17].

### Emotional memory enhancement and consolidation

Emotion has two distinct dimensions, arousal and valence [18,19], both of which influence long-term memory [1,2]. High-arousal stimuli capture attention and are prioritized over low-arousal stimuli at encoding [20,21], sometimes leading to a memory advantage [22]. Valence effects on memory are also common. In younger adults, some studies have shown a memory advantage for negatively valenced material, relative to positive and neutral material [4,23,24], although there have also been reports of superior memory for neutral, compared with emotional, stimuli [6,25–28].

One factor that may account for some of the variability of emotion effects on memory reported in the literature is variation in retention intervals. After encoding, memory traces are thought to require a period of time to stabilize [29–31]. Indeed, some studies have reported an increased memory advantage for emotional material at longer, as compared to shorter, study-test delays, consistent with the idea that emotion modulates long-term memory consolidation [5,13,14,16,22,32–35].

### Mechanisms underlying the emotional modulation of memory

Performance on old-new recognition tests is influenced by memory processes (e.g., encoding, storage, and retrieval) as well as by decision processes that operate on memory representations (e.g., response bias, memory bias). While memory processes have received the most attention in the literature on emotional memory, decision processes are also affected by emotion, and are of particular relevance to the current study.

The bulk of the existing literature on emotion and decision processes of recognition has employed a signal-detection approach [36], involving the analysis of receiver-operating characteristic curves, to separate the effects of emotion on response bias from those on discriminability [6–8]. Some of these studies have suggested that emotion effects on memory reflect more liberal responding for emotional items, rather than improved memory. In Dougal and Rotello’s study of immediate recognition of emotional and neutral words [6], for example, negative stimuli were found to produce a liberal response bias, whereas neither negative nor positive valence affected discriminability. Similar findings were reported by Kapucu and colleagues [8] and, using a similarity choice model [37], by Thapar and Rouder [9]. In a study examining valence and arousal effects, Grider and Malmberg [7] found that both dimensions influenced discriminability. In addition, positive words, but not negative or neutral words, produced a liberal response bias. Arousal did not affect response bias, providing evidence that valence and arousal have distinct effects on cognitive processing.

A limitation of the studies reviewed thus far is their focus on immediate memory tests, which—as noted—may be less sensitive to emotion effects than delayed tests. Additionally, the studies cited above all used verbal materials, which may evoke a more subdued emotional response than pictures [38], and may engage a different set of mnemonic processes [39]. Two more recent studies have addressed these limitations. Using the discrimination index *P*_*r*_ and bias index *B*_*r*_, derived from Two-High Threshold Theory [40], Weymar, Löw, Melzig, and Hamm [41] found enhanced discrimination for negative and positive arousing pictures as compared to neutral, low-arousing pictures after a 1-week study-test lag. They also found that emotional pictures were associated with a more liberal response bias than neutral pictures. A subsequent study [42] examined emotion effects on memory at study-test lags of 1 week and 1 year. Again, emotion enhanced memory at both intervals, although, not surprisingly, overall memory performance was lower at the 1-year compared to the 1-week lag. Additionally, response bias was more liberal for emotional items (both negative and positive), and this effect was smaller after one year. These findings suggest that the length of the retention interval may play a critical role in influencing emotion-induced changes in accuracy but may not influence bias to the same extent.

Most of the literature reviewed thus far has used accuracy-based modeling procedures to examine how emotion affects memory and bias. However, accuracy is in a trade-off relationship with reaction time (RT), and RT data provide a rich source of additional information about emotion effects on memory. To tap into this information, some researchers [10,25,43,44] have used the diffusion model [17], a sequential-sampling model that is well suited to the analysis of binary decision tasks such as old-new recognition. The diffusion model also allows us to access a second type of decision bias, *memory bias—*the relative accessibility of memories—which has largely been ignored in the literature but may be important in understanding emotion modulated memory. As such, we believe that investigations into the influence of emotion and study-test lag on cognitive mechanisms underlying memory are well served by this model. Before we describe the specific aims of the current study, we provide a brief overview of the diffusion model.

### The Diffusion Model

**Fig 1:**
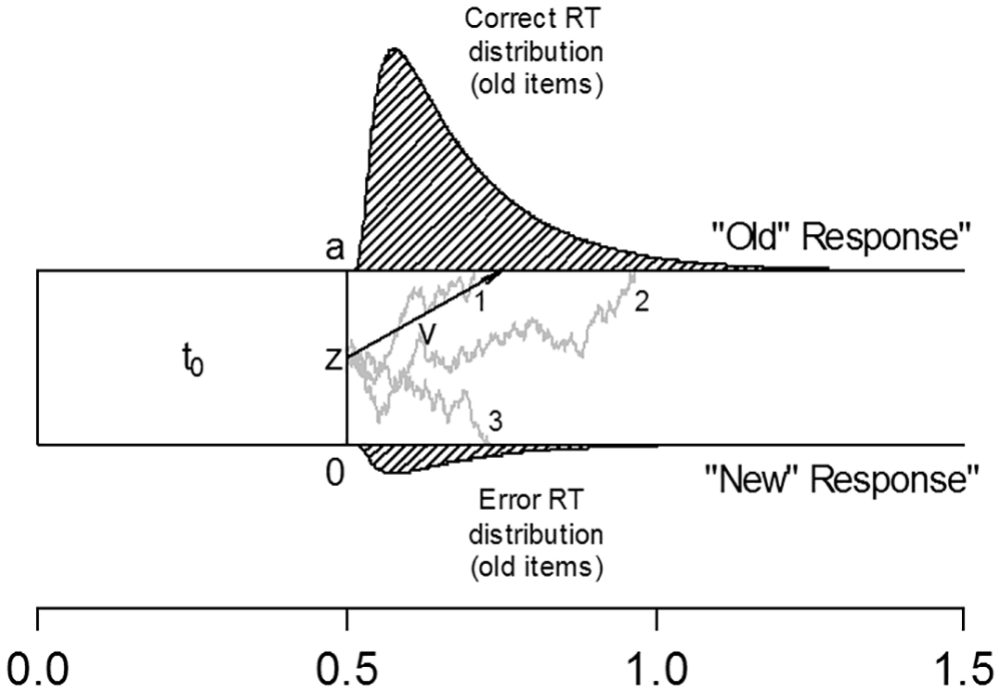
The Diffusion Model [17]. Illustration of the diffusion process for the classification of an “old” item as either “old” or “new”. The decision process starts at point *z* and moves toward the upper boundary or lower boundary by a drift rate ν. In this example, “old” response corresponds to the upper (and correct) boundary *a*, and is driven by a positive drift rate. Three sample paths are illustrated with responses 1 and 2 ending in a correct response at the upper boundary (“old”) but path 3 drifts toward the lower boundary 0, ending in an incorrect response “new”. RT = reaction time; *t*_0_ = perceptual motor RT.

The main assumption of the model is that information is accumulated over time toward one of two decision criteria, and this evidence-accumulation process is noisy. The model takes into account all aspects of the data, including full distributions of correct and error RTs, and the probabilities of correct and error responses [17,45,46]. The diffusion model analysis provides estimates of the processing components thought to underlie the decision process [11,17,45]. Critically, non-decisional processes (perceptual-motor RT; model parameter *t*_*0*_), are separated from decisional processes. For example, Fig 1 illustrates the decision process for two stimulus categories, “old” (upper boundary) and “new” (lower boundary).

The decision process begins at a starting point (parameter *z*) and gradually advances toward one of the two response boundaries. If *z* is closer to one of the two boundaries than to the other, response bias is present. In the current example, if *z* is closer to the upper boundary, the “old” response is favored, and if it is closer to the lower boundary, the “new” response is favored. The starting point can vary from trial to trial, and its variability is captured by parameter *s*_*z*_. The distance between the two boundaries (parameter *a*) indicates the amount of information needed to make a decision. Starting point and boundary separation can be experimentally manipulated. For example, if a particular response is associated with a reward, the starting point tends to move closer to the corresponding boundary [47,48]. If instructions emphasize speed, boundary separation is reduced, resulting in short RTs but low accuracy because the accumulation process is more likely to hit the wrong boundary by mistake. Alternatively, when accuracy is emphasized, boundary separation is large, accuracy is high but RTs are long.

The drift rate (parameter ν) is the average rate at which information accumulates towards either the upper or the lower boundary. Once a boundary is reached, the decision process ends and a response is given (i.e., button press). A positive drift rate indicates that the decision process is being driven toward the upper boundary, as illustrated in the example by the single arrow pointing up. Negative drift rate indicates the process is being driven toward the lower boundary. Drift rate captures the strength or quality of the retrieved information, and is similar to signal detection parameter *d’*. Unlike *d’*, however, drift depends on both accuracy and speed. Steeper (i.e., larger) drift rates are associated with higher accuracy and shorter reaction times. Within-trial variability in drift (i.e., the diffusion constant), illustrated by the jagged lines, contributes to the incidence of error responses, and to variability in finishing times (i.e., RT distributions; [45]). Drift also varies across trials with a standard deviation of s_ν_ (not depicted in the figure).

The non-decision time (*t*_*0*_) sums up the duration of all non-decision processes before and after the decision process (i.e., encoding and response execution, respectively). The model allows non-decision time to vary across trials (variability parameter *s*_*t0*_). Total response time is modeled as the sum of decision time (as predicted by the diffusion process) and non-decision time.

### The current study

The primary goal of the current study was to examine the effects of arousal (high vs. low) and valence (negative vs. positive) on the decision biases affecting delayed recognition. Our approach was novel in a number of ways. First, although time-dependent effects of arousal and valence on memory have been examined previously, no study has investigated the effects of these factors in a single experiment. Second, most previous studies have contrasted emotional with neutral items. Our primary analysis focused on positive and negative materials only, which allowed us to assess the effects of valence and arousal within a relatively homogenous set of emotional items. Furthermore, we used the diffusion model [17] to determine whether emotion and time affect two distinct decision biases: response bias and memory bias [10,11]. This approach was uniquely suited to test two independent hypotheses about possible influences of emotion on decision biases: a motivational (response-bias) hypothesis, and a mnemonic (memory-bias) hypothesis.

### Motivational (response bias) hypothesis

Response bias (*z/a*) is a preference for “old” or “new” responses. In terms of diffusion parameters, it is defined as the placement of the starting point (*z*) relative to the distance of decision boundaries (0 and *a*; see Fig 1). If the starting point is closer to the “old” threshold than to the “new” threshold (*z/a* > 0.5), a liberal response bias is present, whereas the reverse pattern (*z/a* < 0.5) represents a conservative response bias. This response bias measure is conceptually similar to the signal-detection criterion *c* ([49]; see Leite & Ratcliff, 2011 and Wagenmakers, 2009 [50,51] for a discussion on the similarities between signal detection and diffusion model accounts and see White and Poldrack, 2014 [52] for a more detailed account of bias).

We predicted that both valence and arousal would modulate response bias, as has been shown previously in the literature. Response bias is thought to reflect the influence of goals and motivations at the retrieval stage (e.g., Healy & Kubovy, 1978 [53]). Emotional stimuli are motivationally significant because they signal rewarding or aversive experiences (e.g., see Rolls, 2000 [54]). The motivation to prioritize these signals should be stable over time, leading us to predict that response bias would not be influenced by the length of the retention interval (i.e., study-test delay).

### Mnemonic (memory bias) hypothesis

Memory bias is a general tendency, across recognition targets and distractors, to extract mnemonic information from memory that favors either an “old” or a “new” response [10,11]. Signal-detection measures of recognition memory confound response bias and memory bias, making it impossible to distinguish the effects of emotion on the two types of bias. In the diffusion model, memory bias is defined by the position of the drift criterion—the participant’s standard for how strong memory evidence has to be to move toward the top versus the bottom boundary. This type of bias is thus located at the level of memory retrieval processes, rather than at the response level. In terms of diffusion parameters, memory bias is defined as the sum of the drift rates of old and new items (ν_old_ + ν_new_). When familiarity and novelty signals are equally strong, their sum is zero because drift rates for targets are positive while drift rates for distractors are negative (see Fig 1). Memory bias scores above zero indicate familiarity bias, whereas scores below zero indicate novelty bias.

We predicted that high arousal and negative valence would produce familiarity bias, as both factors have been shown to produce mnemonic benefits relative to low arousal and positive valence. Furthermore, we predicted that familiarity bias would increase over time for high-arousal items, but not for negative items. In other words, the arousal effect on memory bias was predicted to interact with study-test lag, whereas the valence effect was expected to remain stable over time. This prediction was made because arousal—but not valence—has been shown to affect the consolidation of memory traces [13,22].

## Method

### Participants

Ninety-seven undergraduate students from Ryerson University participated in return for partial course credit. Participants completed a health questionnaire assessing a history of brain or head injuries, psychiatric illness, use of psychotropic medications, and current depression. Twenty-four participants were excluded because of responses on the health questionnaire or because they failed to return for the second testing session. As a result, the final sample included 73 participants, with 38 participants (7 males) in the 1-day test delay condition and 35 participants (4 males) in the 7-day condition. The median age was 20.4 (range: 18–30 years) in the 1-day group and 20.7 (range: 18–35 years) in the 7-day group.

### Ethics Statement

All procedures were approved by the Research Ethics Board at Ryerson University (REB 2008-123-1). Participants provided written and informed consent.

### Design

The study employed a mixed factorial design that included the between-subjects factor test delay (1-day, 7-day) and the within-subjects factors test status (target, distractor) and arousal (high, low). Valence (positive, negative, neutral) was also manipulated within subjects but was not fully crossed with arousal (see next section and Table 1). There are not enough high-arousal neutral stimuli to be included as a separate category, thus we did not include this class of stimuli in the current experiment.

**Table 1:** Mean Arousal and Valence Ratings of the Experimental Stimuli.

Valence Category	Arousal Category	
	Low-Arousal	High-Arousal
Positive	A: 4.35 (.41)	A: 5.86 (.48)
	V: 7.08 (.64)	V: 7.03 (.60)
Negative	A: 4.91 (.43)	A: 6.08 (.36)
	V: 2.94 (.59)	V: 2.72 (.72)
Neutral	A: 3.59 (.55)	—
	V: 4.92 (.41)	—

Note. A = Mean arousal rating. V = Mean valence rating. Standard deviations are shown in parentheses. No high-arousal neutral items were included. Ratings were taken from published norms [55].

### Stimuli and apparatus

The experimental stimuli included 270 pictures from the International Affective Picture System (IAPS; [55]). The 270 stimuli were selected from a collection of 350 IAPS images used by Bowen and Spaniol [25], and were grouped into five sets of 54 stimuli selected to represent the different combinations of valence and arousal (see Table 1). The sets were equated for semantic content. This was accomplished by classifying the images according to semantic categories (e.g., animals, faces, inanimate objects) and matching the sets on the number of items from each category.

Given the central role of arousal and valence for the rationale of the study, it was important to establish how these properties varied across the stimulus sets. To this end, we conducted a series of analyses of variance (ANOVAs) on arousal and valence ratings.

A 2 (arousal category: high vs. low) x 2 (valence category: negative, positive) ANOVA on *arousal ratings for emotional items* (i.e., excluding neutral items) yielded a main effect of arousal category, such that arousal ratings in the high-arousal category (*M* = 5.97) were higher than arousal ratings in the low-arousal category (*M* = 4.63), *F*(1, 212) = 538.123, *p* < .001, η_p_^2^ = .72. There was also an effect of valence category on arousal ratings, such that arousal ratings for negative items (*M* = 5.5) were higher than those for positive items (*M* = 5.1), *F*(1, 212) = 44.34, *p* < .001, η_p_^2^ = .17. There was also a significant interaction, *F*(1, 212) = 8.80, *p* = .003, η_p_^2^ = .04, such that negative compared to positive items had higher arousal ratings in both the high, *t*(106) = 2.61, *p* = .01, η^2^ = .06, (*M*_*Neg*_ = 6.08, *M*_Pos =_ 5.86) and low-arousal category, *t*(106) = 6.80, *p* < .001, η^2^ = .31, (*M*_*Neg*_ = 4.91, *M*_Pos =_ 4.35).

A 2 (arousal) x 2 (valence) ANOVA on *valence ratings for emotional items* yielded a main effect of valence category, *F*(1, 212) = 2363.82, *p* < .001, η_p_^2^ = 92, such that valence ratings in the negative-valence category (*M* = 2.83) were lower than those in the positive-valence category (*M* = 7.06). There was no main effect of arousal category, nor an Arousal x Valence Category interaction, *F*(1, 212) ≤ 2.19, *p* ≥ .14, η_p_^2^ ≤ .01.

We also conducted one-way ANOVAs of valence category on ratings in the 3 low-arousal categories (left column of Table 1). Valence ratings differed significantly as a function of valence category, *F*(2, 159) = 749.88, *p* < .001, η_p_^2^ = .90, such that valence ratings in the negative category were lower than in the neutral category, and ratings in the neutral category were lower than those in the positive category, *t*(106) ≥ 20.19, *p* < .001, η^2^ ≥ .79. Arousal ratings also differed among the three categories, *F*(2, 159) = 107.14, *p* < .001, η_p_^2^ = .57. Arousal ratings in the negative category (*M* = 4.9) were higher than those in the positive category (*M* = 4.3), and arousal ratings in the positive category were higher than those in the neutral category (*M* = 3.6), *t*(106) ≥ 6.80, *p* < .001, η^2^ = .30.

In summary, the item sets differed on valence and arousal in the required ways. In addition, items in the three valence categories also differed on rated arousal, with negative items being rated as more arousing than positive and neutral items. However, these effects were relatively small.

We divided the stimuli in each cell of Table 1 into two sub-lists (A and B), so that the assignment of specific stimuli to target or distractor status on the recognition test would be counterbalanced across participants. For half of the participants, List A stimuli served as targets and List B stimuli served as distractors, whereas the other half of the participants received the reverse assignment. The lists were again equated for semantic content by assigning roughly equal numbers of exemplars from different semantic categories (e.g., animals, faces, inanimate objects) to each list. When we included List (A, B) as an additional between-item factor in the analyses reported above, we observed no significant main effects of List, nor any interactions.

The experimental tasks were created in E-Prime (Psychology Software Tools, Inc.). Stimulus presentation was controlled by a Tribus desktop with a 19” monitor and a viewing distance of approximately 50 cm. All study and test stimuli appeared in the centre of the screen against a black background.

### Procedure

During the first session, participants were told that the study investigated the effect of emotion on attention. No mention was made of the upcoming memory test. After providing informed consent and filling out the health questionnaire, participants completed the study phase of the experiment. One-hundred thirty-five stimuli (54 high-arousal and 81 low-arousal) were presented in random order, intermixed with twenty-one additional stimuli (also from the IAPS) which served as buffer items and were not included in the analyses. Each trial started with a fixation cross lasting 750 ms, followed by a 500-ms pause and a 3-s stimulus presentation. Participants were asked to view the stimuli passively as if they were watching television. Participants in the 1-day condition returned for the memory test 24 hours after the study session, whereas participants in the 7-day condition returned one week later. During the recognition test, 135 studied targets, 135 unstudied distractors, and 30 buffer items (half old, half new) were presented in random intermixed order. Participants made old-new judgments using the “x” and “,” keys. The key assignment was counterbalanced across participants. Each stimulus remained on screen until a response was made.

## Results

Because the research questions motivating the current study focused on memory for emotional stimuli, neutral items were not included in the first two analyses. Inclusion of neutral items in the arousal analyses would have doubled the number of items in the low-arousal category and likely “artificially” deflated the low-arousal ratings. In order to keep analyses consistent, neutral items were therefore included in the first set of valence analyses. To test the hypotheses about arousal effects (high vs. low) on memory, we collapsed across negative and positive items (i.e., top two rows of Table 1). Likewise, to test the hypotheses about valence effects (positive vs. negative), we collapsed across high-arousal and low-arousal items. This “two-step” analysis approach, alternately collapsing over the valence or the arousal dimension, was necessary because estimating separate diffusion models for all combinations of arousal and valence was not practically feasible (see also the section on ‘limitations and future directions’ in the Discussion).

To facilitate a comparison of our results with previous studies on emotional memory, we also report an analysis of valence and delay effects. This analysis included negative, positive and neutral items of low-arousal only (i.e., the left-hand column of Table 1).

For each analysis, extreme outlier RTs were eliminated using Tukey’s method [56]. Tables 2, 3 and 4 present data after outlier removal. Additional details regarding the treatment of outliers are provided in the description of the diffusion model fitting. For each analysis, the critical results involve the diffusion-model measures (response bias, memory bias), but we also report statistics for other common measures (hit rate, false alarm rate, *d*’, median RT) to facilitate comparison with other studies (see Tables 3 and 4).

**Table 2:** Means of Diffusion Model Parameters for Participants with Good Model Fit.

	1-day	7-day
**Parameter**	**Analysis of Arousal Effects**	
t_0_	.66 (.16)	.74 (.16)
*z/a*__high_	.57 (.12)	.53 (.09)
*z/a*__low_	.62 (.13)	.51 (.10)
ν_old_high_	.69 (.61)	.47 (.47)
ν_old_low_	.48 (.56)	.33 (.34)
ν_new_high_	-.61 (.30)	-.01 (.28)
ν_new_low_	-.57 (.27)	-1.23 (.60)
*s*_*t*_	.27 (.15)	.34 (.23)
*s*_*z*_	.46 (.29)	.47 (.26)
*s*_*ν*_	.55 (.32)	.37 (.26)
*p*	.51 (.24)	.50 (.25)
	**Analysis of Valence Effects**	
t_0_	.65 (.17)	.73 (.16)
*z/a*__neg_	.56 (.09)	.53 (.10)
*z/a*__pos_	.60 (.11)	.59 (.09)
ν_old_neg_	.79 (.70)	.55 (.51)
ν_old_pos_	.48 (.48)	.30 (.41)
ν_new_neg_	-.05 (.22)	-.05 (.23)
ν_new_pos_	-1.35 (.61)	-1.17 (.50)
*s*_*t*_	.27 (.16)	.30 (.16)
*s*_*z*_	.48 (.30)	.47 (.28)
*s*_*ν*_	.60 (.43)	.42 (.29)
*p*	.41 (.28)	.51 (.28)

Note: t_0_ = non-decision time; *z/a* = response bias value (starting point value divided by boundary separation); ν_old_ = drift rate for target items; ν_new_ = drift rate for target items; *s*_*t*_ = non-decision time variability; *s*_*z*_ = starting point variability; *s*_*ν*_ = drift rate variability; *p* = values which indicate goodness-of fit. 1-day = 1-day study-test lag; 7-day = 7-day study-test lag. High = high-arousal; Low = low-arousal; Neg = negative; Pos = positive; Standard deviations are shown in parentheses.

**Table 3:** Arousal and Valence Effects: Means of Signal Detection Parameters and Median Reaction Times for Participants with Good Model Fit.

	HR	FAR	*d’*	RT_Hit_	RT_CR_
**1-day**					
**Arousal Analysis (N = 38)**					
High	.71 (.13)	.30 (.08)	1.14 (.48)	1,266 (577)	1,377 (398)
Low	.64 (.16)	.31 (.05)	.88 (.53)	1,197 (374)	1,276 (378)
**Valence Analysis (N = 37)**					
Negative	.71 (.15)	.49 (.04)	.62 (.45)	1,161 (285)	1,259(272)
Positive	.67 (.13)	.13 (.11)	1.75 (.74)	1.195 (353)	1,240 (280)
**7-day**					
**Arousal Analysis (N = 34)**					
High	.68(.14)	.50 (.07)	.48 (.37)	1,236 (271)	1,372(427)
Low	.61 (.14)	.15 (.10)	1.42 (.45)	1,250 (276)	1,257 (315)
**Valence Analysis (N = 37)**					
Negative	.68 (.13)	.48 (.06)	.55 (.36)	1,259 (272)	1,379 (404)
Positive	.61 (.14)	.18 (.13)	1.36 (.49)	1,240 (280)	1,329 (402)

Note: HR = hit rate; FAR = false alarm rate; *d’* = discriminability index; RT_Hit_ = reaction time for hits; RT_CR_ = reaction time for correct rejections. 1-day = 1-day study-test lag; 7-day = 7-day study-test lag. Standard deviations are in parentheses. Outlier RTs were removed before the calculation of these values.

**Table 4:** Means of Signal Detection Parameters, Median Reaction Times and Mean Diffusion Model Parameters of Low-Arousal Valence Analyses for Participants with Good Model Fit.

Parameter	1-day	7-day
t_0_	.66 (.17)	.69 (.15)
*z/a_neg*	.53 (.12)	.59 (.12)
*z/a_pos*	.56 (.15)	.58 (.11)
*z/a_neu*	.53 (.15)	.52 (.13)
ν_old_neg_	.57 (.78)	.42 (.60)
ν_old_pos_	.41 (.63)	.23 (.48)
ν_old_neu_	.17 (.32)	.01 (.42)
ν_new_neg_	.06 (.24)	-1.21 (.53)
ν_new_pos_	-1.42 (.62)	-1.22 (.69)
ν_new_neu_	-1.47 (.74)	-1.17 (.47)
*s*_*t*_	.25 (.16)	.23 (.12)
*s*_*z*_	.56 (.33)	.52 (.23)
*s*_*ν*_	.46 (.27)	.34 (.21)
HR_neg	.67 (.18)	.65 (.16)
HR_pos	.61 (.17)	.57 (.16)
HR_neu	.55 (.16)	.48 (.15)
FAR_neg	.53 (.06)	.16 (.10)
FAR_pos	.10 (.11)	.16 (.13)
FAR_neu	.10 (.12)	.11 (.09)
*d*’_neg	.44 (.63)	1.51 (.59)
*d*’_pos	1.79 (.78)	1.39 (.60)
*d*’_neu	1.61 (.69)	1.30 (.46)
RT_Hit_neg_	1215 (376)	1265 (356)
RT_Hit_pos_	1222 (366)	1229 (262)
RT_Hit_neu_	1208 (397)	1245 (256)
RT_CR_neg_	1386 (461)	1333 (408)
RT_CR_pos_	1260 (367)	1264 (341)
RT_CR_neu_	1145 (283)	1206 (404)

Note: HR = hit rate; FAR = false alarm rate; *d’* = discriminability index; RT_Hit_ = reaction time for hits; RT_CR_ = reaction time for correct rejections; t_0_ = non-decision time; *z/a* = response bias value (starting point value divided by boundary separation); ν_old_ = drift rate for target items; ν_new_ = drift rate for target items; *s*_*t*_ = non-decision time variability; *s*_*z*_ = starting point variability; *s*_*ν*_ = drift rate variability. 1-day = 1-day study-test lag; 7-day = 7-day study-test lag. Neg = negative; Pos = positive; Neu = neutral. Standard deviations are shown in parentheses. Outlier trials removed.

### Diffusion model fit

Because outlier RTs can significantly bias the parameter estimates of the diffusion model [45,46], extreme responses were excluded using Tukey’s method of outlier detection on log transformed RTs. Tukey’s method leverages interquartile range (IQR = Q3 –Q1) to filter out very small (low outliers = Q1—k*IQR) and very large data points (high outliers = Q3 + k*IQR), and is independent of distributional assumptions, making it ideal for inherently skewed RT data [56]. Further, to utilize as many trials as possible we used a conservative k-value of 2.5 for the Tukey calculation. As a result, trials were removed for 15 participants in the 1-day delay condition and 10 participants in the 7-day delay condition. Each of these 25 participants had an average of 2.71 trials removed (approximately 1% of their total responses). Across participants, 65 trials (0.3% of all trials) were removed in total. The number of trials removed varied as a function of valence, *F*(1, 23) = 5.06, *p* = .03, with more negative trials than positive trials removed. The number of trials removed did not vary significantly as a function of test delay, test status, or arousal, or their interactions, *F* ≤ 3.67, *p* ≥ .07.

Using the fast-dm program [57,58] we estimated diffusion model parameters separately for each participant and experimental condition of interest. By arbitrary assignment, the upper threshold was associated with “old” responses, whereas the lower threshold was associated with “new” responses (see Fig 1). There are two empirical cumulative RT distributions, one for the upper response boundary and one for the lower response boundary. The upper boundary (i.e., old responses) was arbitrarily assigned a positive sign, and the lower boundary (i.e., new responses) was assigned a negative sign. The Kolmogorov-Smirnov (KS) test statistic was used to estimate model fit between the predicted and empirical RT distributions [48]. A significant KS statistic (p-value parameter < .05) indicates that the maximal vertical distance between the two RT distributions is large, indicating poor model fit.

To test the hypotheses about arousal effects, we collapsed data across positive and negative valence. Separate models were estimated for each participant and test delay. Of particular interest were drift rates and starting point, because these parameters affect the critical measures (response bias, and memory bias). Separate drift rates (ν) were estimated for targets and distractors at each level of arousal (high, low). Separate starting point (*z*) and boundary separation (a) values were estimated at each level of arousal. This resulted in the estimation of 2 target drift rates, 2 distractor drift rates, 2 starting points, and 2 boundary separation values per participant. The remaining parameter—non-decision time (*t*_*o*_), and variances in non-decision time (*s*_*t*_), starting point (*s*_*z*_), and drift rate (*s*_*v*_)—were constrained to be constant over conditions. In total, 12 parameters were estimated per participant at each test delay.

A similar logic was used to estimate models to test hypotheses about valence effects. Again, separate models were estimated for each test delay (1-day vs. 7-day). Separate drift rates were estimated for targets and distractors at both levels of valence (negative vs. positive). Separate starting point (z) and boundary separation (*a*) values were estimated at each level of valence. Non-decision time (*t*_*o*_), and variance in non-decision time (*s*_*t*_), starting point (*s*_*z*_) and drift rate (*s*_*v*_) were not estimated separately for each level of valence but were constrained to be equal across experimental conditions. In total, 12 parameters were estimated per participant at each test delay. Group-level descriptive statistics of the diffusion model parameters are presented in Table 2. Inferential statistics will be reported only for the hypothesis-relevant measures of response bias and memory bias.

Model fit was assessed with a Monte-Carlo simulation [57]. Only participants with good diffusion-model fit were included in the analyses of model-based measures. Additional analyses were run that included all participants (including those with poor model fit) and the pattern of results remained the same. For the analyses of arousal effects, of the 73 participants 3 had poor fit (all in the 7-day condition), leaving 38 and 32 participants in the 1-day and 7-day conditions, respectively. For the analyses of valence effects, 2 of the 73 participants had poor model fit (1 in the 1-day and 1 in the 7-day delay conditions). This left 37 participants in the 1-day delay and 34 in the 7-day delay condition, respectively. In the supporting information S1–S4 Figs, model fit is displayed graphically for each of the conditions.

### Emotional items: Effects of arousal and delay

#### Discriminability and RT

We conducted a 2 x 2 repeated-measures ANOVA on *d*’ [36] with test delay (1-day, 7-day) as a between-subjects variable and arousal (high, low) as a within-subjects variable. There was no main effect of delay, *F*(1, 68) = .37, *p* = .54, η_p_^2^ = .01. The main effect of arousal, *F*(1, 68) = 36.04, *p* < .001, η_p_^2^ = .35, was qualified by a significant Arousal x Delay interaction, *F*(1,68) = 111.42, *p* < .001, η_p_^2^ = .62. Follow-up comparisons revealed that *d*’ was higher for high-arousal stimuli than for low-arousal stimuli at the 1-day delay, t(37) = 4.75, *p* < .001, η^2^ = .38, but the reverse was true at the 7-day delay, t(31) = 8.90, *p* < .001, η^2^ = .72. See Table 3 for the means for each condition.

A 2 x 2 repeated-measures ANOVA on median RTs, separately for hits and correct rejections, with study-test delay (1-day, 7-day) as the between subjects variable and arousal (high, low) as the within-subjects variable. For hits, there were no significant effects of either variable, nor a significant interaction, *F*(1, 68) ≤ 1.86, *p* ≥ .18. For correct rejections, there was a significant main effect of arousal, *F*(1, 68) = 21.37, *p* < .001, η_p_^2^ = .24, such that participants responded faster to low-arousal (*M* = 1266 ms) compared to high-arousal (*M* = 1374 ms) distractor items. Neither the interaction with delay, nor the main effect of delay, was significant, *F*(1, 68) ≤ .09, *p* ≥ .77.

#### Response bias

Response bias was defined as *z/a*, that is, as the relative placement of the starting point between the two response boundaries. Values above .5 indicate a liberal or “old” bias, and values below .5 a conservative or “new” bias.

**Fig 2:**
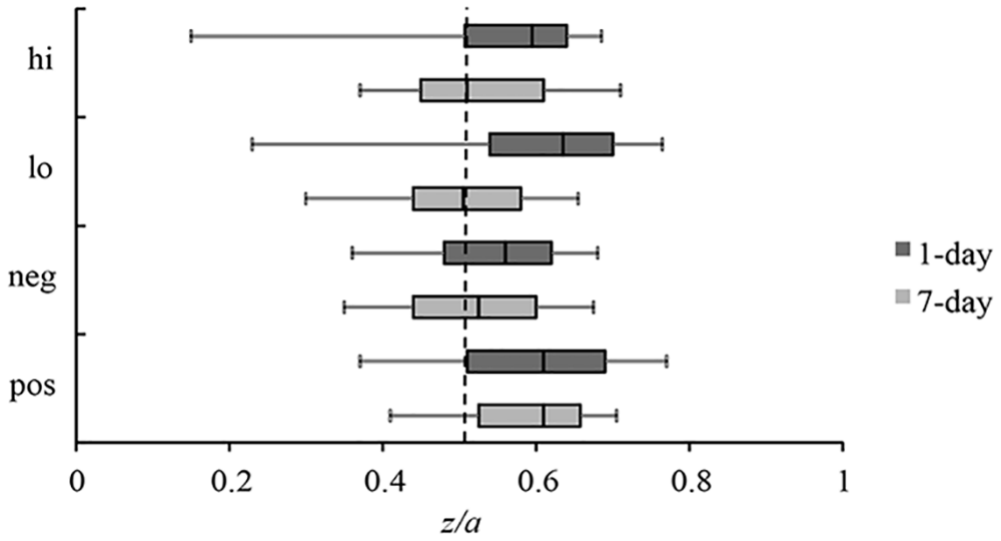
Response Bias (z/a). Box plots of the distribution of response bias values for high and low-arousal items and negative and positive items at each lag. The line in each box represents the median. Response bias values above .5 (to the right of the dotted line) indicate a bias to classify items as “old”, whereas values below .5 indicate a bias to classify items as “new”. Error bars represent standard error. Hi = high-arousal items; Lo = low-arousal items; Neg = negative items; Pos = positive items; 1-day = 1-day study-test lag; 7-day = 7-day study-test lag.

The response bias indices are shown in Fig 2.There was significant main effect of arousal and delay, *F*(1, 68) ≥ 5.75, *p* ≤ .01, η_p_^2^ ≥ .08, qualified by a significant Arousal x Delay interaction, *F*(1, 68) = 21.49, *p* < .001, η_p_^2^ = .24. Follow-up paired *t*-tests indicated that low-arousal items produced a more liberal response bias at the 1-day delay, *t*(37) = 4.50, *p* < .001, η_p_^2^ = .35, but at the 7-day delay, there was a statistical trend in the reverse direction, *t*(31) = 2.34, *p* = .05, η_p_^2^ = .11. One-sample *t*-tests showed that response bias for high-arousal and low-arousal items was significantly greater than .5 (no bias) at the 1-day delay, *t*(37) ≥ 3.80, *p* < .001, η^2^ ≥ .28, but response bias for high and low-arousal items was not significantly different from .5 at the 7-day delay, *t*(31) ≤ 1.67, *p* ≥ .11, η^2^ ≤ .08.

#### Memory bias

Memory bias was operationalized as ν_old_ + ν_new_. Positive values indicate a familiarity bias, that is, a tendency to extract information favoring an “old” response, independent of discriminability. Negative values indicate a novelty bias, that is, a tendency to extract information in favor of a “new” response, independent of discriminability.

**Fig 3:**
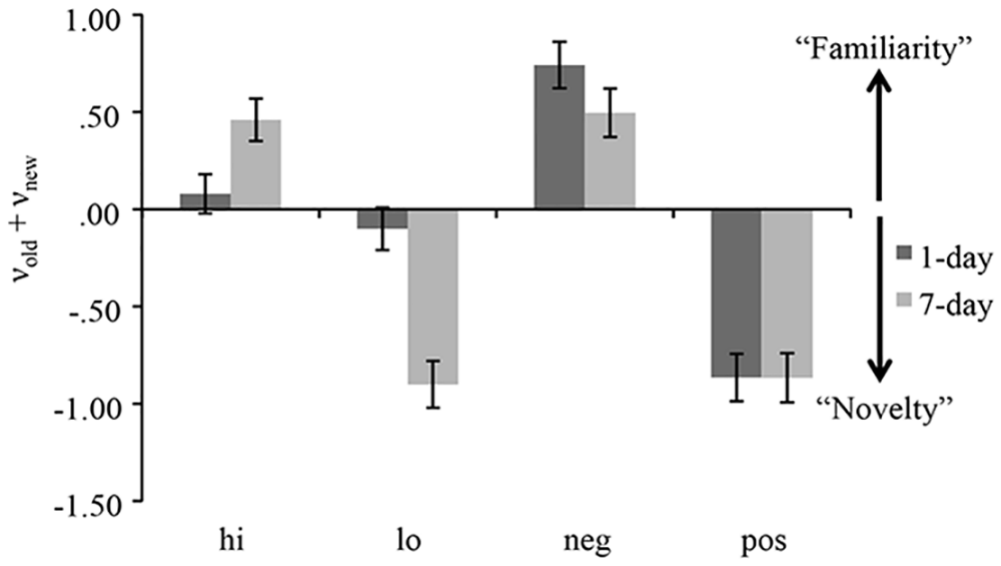
Memory Bias (ν_old_ + ν_new_). Mean memory bias values for high and low-arousal items, and negative and positive items at each lag. Positive memory bias values indicate familiarity bias, whereas negative values indicate novelty bias. Error bars represented the standard errors. Hi = high-arousal items; Lo = low-arousal items; Neg = negative items; Pos = positive items; 1-day = 1-day study-test lag; 7-day = 7-day study-test lag.

Memory bias indices are shown in Fig 3. There was a marginally significant main effect of study-test delay, *F*(1, 68) = 2.92, *p* = .09, η_p_^2^ < .001 and a significant main effect of arousal, *F*(1, 68) = 75.22, *p* < .001, η_p_^2^ = .53, qualified by a significant Arousal x Delay interaction, *F*(1, 68) = 44.10, *p* < .001, η_p_^2^ = .39. Follow-up comparisons revealed that memory bias indices were more negative for low-arousal items than for high-arousal items. This difference was marginally significant at the 1-day delay, *t*(37) = 1.70, *p* < .09, η^2^ < .001, and it was significant at the 7-day delay, *t*(31) = 9.27, *p* < .001, η^2^ = .73. Post-hoc one-sample *t-*tests revealed that at the 1-day delay, memory bias for high-arousal and low-arousal items did not differ significantly from zero (no bias), *t*(37) ≤ .77, *p* ≥ .33, η^2^ ≤ .003. At the 7-day delay, the memory bias value was significantly greater than zero for high-arousal items, *t*(31) = 4.50, *p* < .001, η^2^ = .39, and significantly below zero for low-arousal items, *t*(31) = 7.30, *p* < .001, η^2^ = .63.

### Emotional items: Effects of valence and delay

#### Discriminability and RT

A 2 x 2 ANOVA on *d’* was conducted with valence (negative, positive) as the within-subjects variable. There was a main effect of delay, *F*(1, 69) = 5.24, *p* = .03, η_p_^2^ = .07, and a main effect of valence, *F*(1, 69) = 176.44, *p* < .001, η_p_^2^ = .72. These effects were qualified by a significant interaction, *F*(1, 69) = 4.44, *p* = .04, η_p_^2^ = .06. Follow-up comparisons revealed that *d*’ was higher for positive items compared to negative items at the 1-day delay, *t*(37) = 10.87, *p* < .001, η^2^ = .77. The effect was also present—though slightly diminished—at the 7-day delay, *t*(33) = 7.95, *p* < .001, η^2^ = .66.

A 2 x 2 repeated-measures ANOVA was conducted on median RTs, separately for hits and correct rejections, with study-test delay as the between-subjects variable and valence (negative, positive) as the within-subjects variable. For hits, there were no significant effects of either variable, nor a significant interaction, *F*(1, 69) ≤ 2.16, *p* ≥ .15. For correct rejections, there was a significant main effect of valence, *F*(1, 69) = 12.92, *p* = .001, η_p_^2^ = .16, such that participants were faster at responding to positive (*M* = 1314 ms) compared to negative items (*M* = 1394 ms).There was no main effect of delay, nor a significant interaction, *F*(1, 68) ≤ 1.90, *p* ≥ .17.

#### Response bias

Response bias indices are shown in Fig 2. There was a significant main effect of valence, *F*(1, 69) = 17.98, *p* < .001, η_p_^2^ = .21, such that response bias was more liberal for positive compared to negative items. There was no significant main effect of delay and no significant Valence x Delay interaction, *F*(1, 69) ≤ .83, *p* ≥ .37, η_p_^2^ ≤ .01. One-sample *t*-tests showed that response bias values for positive and negative items were significantly greater than .5, *t*(70) ≥ 3.49, *p* ≤ .001, η^2^ ≥ .15.

#### Memory bias

Memory bias indices are shown in Fig 3. The main effect of valence was significant, *F*(1, 69) = 150.40, *p* < .001, η_p_^2^ = .69, such that memory bias values for negative items were more positive than memory bias values for positive items. The main effect of delay was not significant, nor was the Valence x Delay interaction, *F*(1, 68) ≤ 1.30, *p* ≥.31, η_p_^2^ ≤ .02. Follow-up one-sample *t*-tests showed that negative items elicited significant familiarity bias, *t*(70) = 7.14, *p* < .001, η^2^ = .42, whereas positive items elicited significant novelty bias, *t*(70) = 9.94, *p* < .001, η^2^ = .56.

### Low-arousal emotional and neutral items: Effects of valence and delay

To examine the emotional memory advantage and compare to previous work with neutral stimuli, all analyses were run with low-arousal stimuli (left column of Table 1) comparing negative, positive and neutral items. One participant in the 1-day delay condition had poor model fit and is excluded from the results presented below. All mean values of the diffusion model as well as hit rate, false alarm rate, *d’* and median reaction times are presented in Table 4.

#### Discriminability and RT

There was no significant main effect of delay on *d*’, *F*(1, 70) = 1.02, *p* = .34, η_p_^2^ = .01, but there was a significant main effect of valence, *F*(2, 140) = 37.46, *p* < .001, η_p_^2^ = .35, qualified by a significant Valence x Delay interaction *F*(2, 140) = 60.10, *p* < .001, η_p_^2^ = .40. A follow-up one-way ANOVA at the 1-day delay revealed a significant valence effect, *F*(2, 72) = 97.71, *p* < .001, η_p_^2^ = .73. Follow-up *t*-tests indicated that *d’* was significantly lower for negative items (*M* = .44) than for positive (*M* = 1.79), and neutral items (*M* = 1.61), *t*(36) ≥ 10.81, *p* < .001, η^2^ ≥ .76, but *d’* values were only marginally different for positive and neutral items, *t*(36) = 1.90, *p* = .07 η^2^ = .09. A follow-up one-way ANOVA at the 7-day lag indicated no significant effect of valence, *F*(2, 68) = 1.87, *p* = .16, η_p_^2^ = .05.

Separate ANOVAs were carried out on median RTs for hits and correct rejections (see Table 4). For hits, there were no significant effects of either variable, or a significant interaction, *F* ≤ 1.8, *p* ≥ .68. For correct rejections, the main of valence was significant, *F*(2, 140) = .22.11, *p* < .001, η_p_^2^ = .24, but there was no main effect of delay nor a significant interaction *F* ≤ 2.17, *p* ≥ .12, η_p_^2^ ≤ .03. Follow-up *t*-tests for the main effect of valence indicated that RTs were faster for neutral stimuli (*M* = 1174) than for positive (*M* = 1262) and negative (*M* = 1360), *t*(71) ≥ 3.85, *p* < .001, η^2^ ≥ .17, and positive were faster than negative *t*(71) = 3.29, *p* = .002, η^2^ = .13.

#### Response bias

A 2 X 3 ANOVA on response bias values indicated a significant main effect of valence, *F*(2, 140) = 3.75, *p* = .03, η_p_^2^ = .05, but no significant main effect of delay, nor a significant Valence x Delay interaction, *F* ≤ 2.02, *p* ≥ .14. Pairwise comparisons revealed that response bias indices were significantly higher for emotional items (both positive and negative) than for neutral items, *t*(71) ≥ 2.13, *p* ≤ .04, η^2^ ≥ .06. One-sample *t*-tests indicated that response bias values were significantly greater than .5 for emotional items, *t*(71) ≥ 4.23, *p* < .001, η^2^ ≥ .20, but that neutral items did not significantly differ from .5, *t*(71) = 1.47, *p* = .15.

#### Memory bias

A 2 x 3 ANOVA on memory bias indices revealed a significant main effects of valence, *F*(2, 140) = 51.65, *p* < .001, η_p_^2^ = .43, and delay, *F*(1, 70) = 10.78, *p* = .002, η_p_^2^ = .13, qualified by a significant Valence x Delay interaction, *F*(2, 140) = 26.00, *p* < .001, η_p_^2^ = .27. A follow-up repeated measures ANOVA at the 1-day delay revealed a significant valence effect, *F*(2, 72) = 67.99, *p* < .001, η_p_^2^ = .65. Follow-up *t-*tests indicated that memory bias values for negative items (*M* = .63) were significantly different from those for positive items (*M* = -1.04) and neutral items (*M =* -1.32), *t*(37) ≥ 9.63, *p* < .001, η^2^ ≥ .71, but the difference between positive and neutral was only marginally significant, *t*(37) = 1.82, *p* = .08. One-sample *t*-tests indicated that all values were significantly different from zero, *t*(37) ≥ 4.68, *p* < .001, η^2^ ≥ .37. The follow-up repeated measures ANOVA at the 7-day delay, indicated that the effect of valence was only marginally significant, *F*(2, 68) = 2.83, *p* = .07, η_p_^2^ = .08, (*M*_neg_ = -.79, *M*_pos_ = -.99 *M*_neu_ = -1.17).

## Discussion

The goal of this study was to examine how decision biases involved in recognition are affected by emotional arousal and valence over time. During study, participants viewed emotional stimuli that varied in arousal (high vs. low) and valence (negative, neutral, positive). Half of the participants completed an old-new recognition test 24 hours post-encoding, whereas the other half completed the test one week after encoding. Discriminability and RT data for emotional items were submitted to diffusion model analyses to capture the effects of arousal (high, low), valence (negative, positive), and study-test lag (1-day, 7-day) on two outcomes of theoretical significance: response bias, and memory bias. The data support our hypotheses about the effects of emotion and study-test lag on memory bias, but predictions regarding response bias were only partially supported. Before discussing the findings of the diffusion model in more detail, we discuss the results of the discriminability and RT measures. We also provide a separate discussion of valence analyses that incorporate only low-arousal stimuli after the main analyses involving high and low-arousal stimuli.

### Discriminability and RT

The analyses of the signal-detection measure of discriminability (*d’*) revealed that both valence and arousal interacted with study-test delay. Discriminability was enhanced for high-arousal compared to low-arousal items, but only at the short delay. Further, discriminability was enhanced for positive compared to negative items at both study-test lags, but overall discriminability decreased over time for both positive and negative stimuli. At first glance, these findings appear to be at odds with prior reports of emotional enhancement of memory at extended delays of 1 week [35,41] or longer [32,34]. However, in those earlier studies, valence and arousal contributions were not assessed separately, and emotional enhancement was defined relative to a neutral (non-valenced, low-arousal) baseline. In contrast, the principal analyses of the current study focused on emotional items, which allowed for a relatively fine-grained look at the impact of valence and arousal on delayed recognition. The results do converge with prior literature indicating that emotional arousal and valence have dissociable effects on discriminability [59,60] (see Kensinger, 2004 [13], for a review).

Median RT analyses revealed main effects of arousal and valence, but they were not influenced by study-test lag. Responses were slower for high-arousal compared to low-arousal items, and for negative compared to positive items. It is clear that reaction time patterns do not easily map on to patterns associated with accuracy. Furthermore, response bias and memory bias cannot be assessed on the basis of accuracy and RT which do not allow for the separation of motivational and mnemonic influences. The parameters of the diffusion model allow us to look more specifically at these measures. These results are discussed next.

### Response bias

The preference to choose one response over another was defined as the placement of the starting point parameter relative to the decision boundary (*z*/*a*). A value greater than .5 refers to a liberal (i.e., “old”) response bias, whereas a value less than .5 indicates a conservative (i.e., “new”) response bias. According to the motivational hypothesis, the emotional goals that may induce a tendency to endorse arousing and valent stimuli as old should not change as function of time. This prediction was not fully supported. Contrary to the motivational hypothesis, arousal-based modulation of response bias *was* affected by retention interval. We found that low-arousal items elicited a more liberal response bias than high-arousal items at the short retention interval, but at the longer retention interval these values did not significantly differ from each other. Indeed, after 1 week, neither low-arousal nor high-arousal gave rise to significant response bias. This mirrors the finding of Grider and Malmberg [7], who also found no influence of arousal on response bias at a delayed retention interval. Similarities to this earlier study were also found with respect to valence effects on response bias. Specifically, in line with findings from Grider and Malmberg [7], positive valence was associated with a more liberal response bias than negative valence. Furthermore, as predicted, valence did not interact with the length of the retention interval.

Overall, these findings suggest that response bias may be sensitive to both delay-dependent mnemonic factors and delay-independent motivational factors. Liberal responding to emotional arousing and emotionally valent stimuli may be the result of a heuristic strategy (“I was shown several pictures of violent scenes, so any pictures of violent scenes are probably old”). Lack of an interaction with delay suggests that response bias is relatively stable over time when assessing valence, but may be modulated by the neural mechanisms underlying consolidation processes when examining influence of arousal. It is possible response bias is not only at the level of motivational goals but involves more memory processes that for arousal, but not valence, require consolidation. A future study assessing immediate and delayed recognition of arousing and valence stimuli is necessary to answer this question.

### Memory bias

To examine bias at the level of memory retrieval, we combined diffusion drift for targets and distractors (ν_old_ + ν_new_). Positive values of this measure indicate more efficient retrieval of evidence in favor of an “old” response (familiarity bias), whereas negative values indicate more efficient retrieval of evidence in favor of a “new” response (novelty bias). According to the mnemonic hypothesis, the effect of arousal (but not of valence) on memory bias should increase with delay, due to arousal's influence on consolidation processes. This hypothesis was supported. Specifically, high-arousal stimuli produced a familiarity memory bias and low-arousal items a novelty bias and this pattern became pronounced as study-test delay increased, driving the Arousal x Lag interaction and consistent with prior observations in the literature [10,43]. With respect to valence influences on memory bias, positive items elicited novelty bias and negative items gave rise to a familiarity bias at both retention intervals. Taken together, these findings indicate that, over time, high arousal and negative valence provide a mnemonic familiarity advantage over low arousal and positive valence. Additionally, in accordance with our hypothesis, arousal but not valence interacted with retention interval as arousal specifically, has been shown to modulate the memory trace.

### Low-arousal emotional and neutral items

Although the primary goal of the current study was to characterize strictly emotional influences on recognition memory, we also tested the effects of valence with the inclusion of neutral stimuli. Only low-arousal stimuli were included in these analyses to minimize differences in average arousal for positive, neutral, and negative stimulus sets. As noted, even within the low-arousal stimulus set, there remained significant differences in average arousal among the valence categories (negative > positive > neutral). However, the pattern emerging from these analyses is quite distinct from the pattern in the arousal analyses, lending confidence that the results indeed captured valence-specific effects. Overall, the results did not reveal anything additional about emotional memory that was not reported above, but it is clear that emotional memory and the cognitive biases underlying it are different from processes engaged during memory for neutral items. Perhaps not surprising, results for accuracy and RT revealed that sensitivity for neutral items was lower than for emotional items (at the 1-day delay), but correct rejection RTs were faster than for emotional items. The diffusion model results indicated that across retention intervals, neutral items did not elicit a response bias in either direction, but emotional items were associated with a liberal response bias as reported in the results above. One interesting finding was that neutral items elicited a novelty bias, even more so than positive items, and negative items again induced a familiarity bias at the 1-day delay. At the 7-day delay, all three valence categories were associated with a novelty bias. This finding in particular goes against an arousal explanation for these differences in valence, as negative items had the highest arousal ratings but these results do not match the pattern of results from the high and low-arousal analyses.

### Limitations and future directions

Diffusion modeling requires relatively large numbers of observations in each experimental condition [46], and it was important to match the stimulus sets on semantic features [61]. These constraints made it impossible to fully equate the positive, neutral, and negative low-arousal stimulus sets on average rated arousal. Second, within the emotional stimuli, a significant difference in average arousal was present between the valence categories (negative > positive). Ideally, these items sets would have been matched on arousal to avoid potential confounds. However, the pattern emerging from the valence analyses was quite distinct, and in the opposite direction from that obtained in the arousal analyses, lending confidence that the results of the analyses indeed captured valence-specific and arousal-specific effects. For example, memory sensitivity was better for high-arousal compared to low-arousal items, and for positive compared to negative items—even though negative items were significantly more arousing than positive items. Third, our two-step analysis approach did not permit an analysis of potential interactions of valence and arousal on memory for emotional items. This was again related to the difficulty of fitting the diffusion model to noisy individual-participant data. Fitting separate models to each cell of the design (see Table 1) instead of collapsing across cells for the analyses of arousal and valence effects, respectively, would have further increased the proportion of participants with poor model fit in the current study. The investigation of Arousal x Valence interactions thus remains a challenge for future research. Fourth, we did not include an immediate test of memory and therefore cannot make conclusions about the effects of consolidation per se. Future work examining the effects of emotion on decision biases before and after consolidation is an important comparison for future research. Finally, we included neutral stimuli even though the critical hypotheses and analyses focused on positive and negative items only, and we presented all three valence categories in mixed trial blocks. Considering the possibility of “bleed-over” valence effects during encoding, future work should test whether the current findings would hold if positive and negative items were presented in blocked, rather than intermixed, fashion during the study phase.

### Conclusions

Understanding how emotion influences memory is important because so many of our memories have an affective tone, and many affective disorders are associated with memory bias toward highly arousing and negative experiences. The diffusion model can improve analysis of two-choice tasks often employed when investigating differences in healthy and patient populations—such as individuals with PTSD, depression and anxiety—by decomposing accuracy and RT distributions into distinct components of processing to better inform cognitive training [11]. The role of retention interval is particularly important given the constructive nature and malleability of memory made particularly salient in the false memory and eyewitness testimony literature (e.g., Loftus [62]).

The current study is the first to separate the effect of arousal from that of valence on the delayed recognition of incidentally encoded pictures. We utilized the diffusion model to examine two decision biases: response bias and memory bias. There are two novel findings from the study. First, while both arousal and valence lead to a liberal response bias which has been reported previously, we found that the motivations that influence these response preferences at retrieval are stable across long study-test delays for valent stimuli, but bias is reduced for arousing stimuli after a longer delay. Second, memory bias analyses indicated that arousal and valence differentially influence familiarity and novelty signals on memory decisions. In particular, high arousal and negative valence boost familiarity signals relative to low arousal and positive valence, respectively. These findings confirm a “special role” of high arousal and negative valence in emotion-cognition interactions in younger adults [4,23,24] and the impact of arousal, rather than emotional valence on consolidation processes during long-term memory. The dissociation of arousal and valence effects on memory bias illustrates the utility of diffusion modeling for the study of emotion-cognition interactions (see also [11,63]). By jointly analyzing accuracy and RT data and providing individual measures of model fit, diffusion modeling provides a powerful alternative to accuracy-based measures commonly used in the literature on emotional memory which cannot distinguish between response bias and memory bias.

The memory bias findings are particularly interesting because the pattern is quite different from the pattern that emerged for accuracy and reaction time. For example, accuracy was better, and reaction times were shorter, for positive compared to negative items. However, the memory bias results suggest that positive items elicit novelty signals rather than familiarity signals. Thus, participants were more efficient in detecting positively-valenced new stimuli than in recognizing positively-valenced old stimuli. We think this contradiction speaks to the need to examine accuracy and reaction time in a model that allows the inclusion of both simultaneously and how diffusion modeling is particularly useful for examining how emotion affects retrieval dynamics, an issue that has received little empirical study to date.

## Supporting Information

S1 Fig Model Fit for Arousal at 1-day Delay. Fit of the model predictions for response time quartiles and accuracy values for high and low arousal at the 1-day delay.
(TIF)

S2 Fig Model Fit for Arousal at 7-day Delay. Fit of the model predictions for response time quartiles and accuracy values for high and low arousal at the 7-day delay.
(TIF)

S3 Fig Model Fit for Valence at 1-day Delay. Fit of model predictions for response time quartiles and accuracy values for negative and positive valence at the 1-day delay.
(TIF)

S4 Fig Model Fit for Valence at 7-day Retention. Fit of model predictions for response time quartiles and accuracy values for negative and positive valence at the 7-day delay.
(TIF)
