# Supplementary figures and images for: A Diffusion Model Analysis of Decision Biases Affecting Delayed Recognition of Emotional Stimuli
Source: PLoS One. 2016 Jan 19;11(1):e0146769. doi: 10.1371/journal.pone.0146769 (PMC4718681; doi:10.1371/journal.pone.0146769)

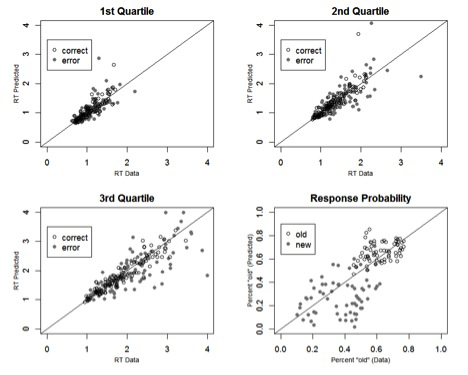

Supplement: S1 Fig — Fit of the model predictions for response time quartiles and accuracy values for high and low arousal at the 1-day delay. (TIF) [file pone.0146769.s001.tif]

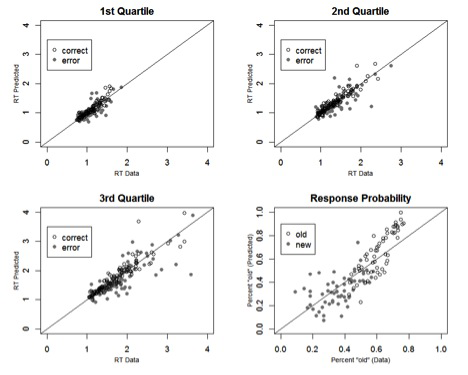

Supplement: S2 Fig — Fit of the model predictions for response time quartiles and accuracy values for high and low arousal at the 7-day delay. (TIF) [file pone.0146769.s002.tif]

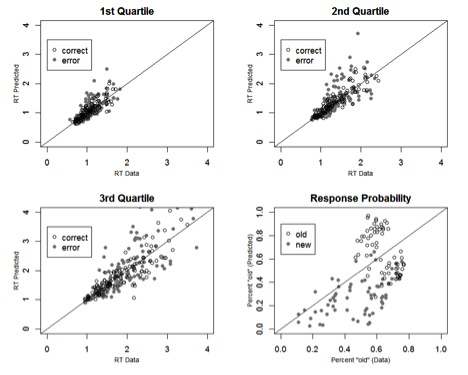

Supplement: S3 Fig — Fit of model predictions for response time quartiles and accuracy values for negative and positive valence at the 1-day delay. (TIF) [file pone.0146769.s003.tif]

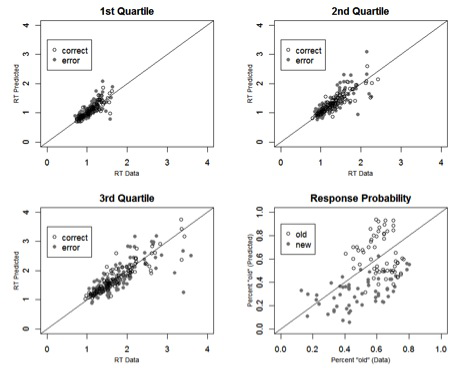

Supplement: S4 Fig — Fit of model predictions for response time quartiles and accuracy values for negative and positive valence at the 7-day delay. (TIF) [file pone.0146769.s004.tif]
